# Editorial: NEUROTRAUMA: From Emergency Room to Back to Day-by-Day Life

**DOI:** 10.3389/fneur.2018.00776

**Published:** 2018-09-19

**Authors:** Renato Anghinah, Wellingson Silva Paiva, Tiago Henrique Falk, Felipe Fregni

**Affiliations:** ^1^Department of Neurology, Universidade de São Paulo, São Paulo, Brazil; ^2^Centre Énergie, Matériaux, Télécommunications, Institut National de la Recherche Scientifique, Quebec, QC, Canada; ^3^Harvard Medical School, Boston, MA, United States

**Keywords:** traumatic brain injury, concussion, diffuse axonal injury, cognitive impairment, treatment

Traumatic brain injury (TBI) is a nondegenerative and non-congenital insult to the brain from an external mechanical force that can lead to permanent or temporary impairment of cognitive, physical, and psychosocial functions (1). TBI is considered a “silent epidemic” not only due to its magnitude, but also because it affects mostly young and productive individuals (2).

Most patients with TBI return home after the critical phase of hospital management. Although some patients manage to regain some degree of independence in their self-care, many are still incapable of applying critical thinking to decision-making processes, providing for their family needs, or continuing work, school or social activities. Moreover, many also manifest mood alterations and depression. As such, patient rehabilitation after hospital discharge is a critical step in returning to their day-by-day lives (3).

The objective of this *Frontiers in Neurology* Research Topic is to present the latest findings and views regarding the pathophysiology and treatment of TBI. It is comprised of 10 papers, each offering a unique view and understanding of how TBI can be detected and managed from the emergency room to back to day-by-day life.

Hayashi et al. evaluated the cortical excitability during the chronic phase of TBI in victims diagnosed with diffuse axonal injury (DAI). Amorim et al. in turn, studied the effects of transcranial direct current stimulation (tDCS) in patients with persistent post-concussion syndrome who demonstrated cognitive deficits in long-term episodic memory, working memory, and executive function following mild TBI. Hashim et al. used diffusion tensor imaging to investigate the apparently normal white matter (assessed by routine magnetic resonance imaging) in the brains of subjects with sub-acute and chronic TBI. Oliveira et al. studied the usefulness of transcranial color-coded duplex sonography for evaluating TBI patients. Vieira et al. described the outcome for patients with a primary diagnosis of DAI 6 months after trauma and identified sociodemographic and clinical factors associated with mortality and dependence at this time point. Dambinova et al. hypothesized that neurotoxicity AMPA, NMDA, and kainite receptor biomarkers might be utilized as part of a comprehensive approach to concussion evaluations. Popovic et al. in turn, described a case of a man with non-traumatic spinal cord injury that was submitted to functional electrical stimulation therapy to restore voluntary reaching and/or grasping function of his hand. Khong et al. conducted a systematic review regarding the evidence for the use of diffusion tensor imaging parameters in the human brain as a diagnostic tool for and predictor of post-concussion syndrome after a mild traumatic brain injury. Kirmani et al. reviewed the literature to understand the role Lastly, of anticonvulsants in the treatment of posttraumatic epilepsy. Rodrigues et al. conducted a review of existing literature regarding the effects of soccer heading on brain structure and function.

## Author contributions

All authors listed have made a substantial, direct and intellectual contribution to the work, and approved it for publication.

### Conflict of interest statement

The authors declare that the research was conducted in the absence of any commercial or financial relationships that could be construed as a potential conflict of interest.
